# Indigenous 2SLGBTQIA+ Identities and Age-Related Cognitive Decline: A Scoping Review

**DOI:** 10.3390/ijerph23060735

**Published:** 2026-05-30

**Authors:** Keith D. King, Skye Wilson, Letebrhan Ferrow, Lane Bonertz, Jessy Dame, Megan Kennedy, Jennifer D. Walker

**Affiliations:** 1 Faculty of Nursing, University of Alberta, Edmonton, AB T6G 1C9, Canada; mrkenned@ualberta.ca; 2 Community Based Research Centre–2Spirit Program, Vancouver, BC V6Z 2H2, Canada; skye.wilson@cbrc.net (S.W.); lane.bonertz@cbrc.net (L.B.); jessy.dame@cbrc.net (J.D.); 3 Department of Health Research Methods, Evidence, and Impact, Faculty of Health Sciences, McMaster University, Hamilton, ON L8N 3Z5, Canada; ferrowl@mcmaster.ca (L.F.); jennifer.walker@mcmaster.ca (J.D.W.)

**Keywords:** Two-Spirit, indigenous, LGBTQ, dementia, cognitive decline, aging

## Abstract

**Highlights:**

**Public Health Relevance—How does this work relate to a public health issue?**

- Age-related cognitive decline is a significant issue as the population of people living longer continues to grow and the number of Indigenous and 2SLGBTQIA+ seniors is also growing.
- Indigenous health is under-researched, and there is even less research on 2SLGBTQIA+ Indigenous Peoples.

**Public Health Significance—Why is this work of significance to public health?**

- Our scoping review provides a foundation for further research in the topic of Two-Spirit, Indigenous, and LGBTQIA+ communities as they age.
- Two-Spirit and Indigenous LGBTQIA+ individuals hold unique gifts, medicines, and experiences that have yet to be researched in a significant way, providing opportunities for enhancing public health interventions across the lifespan.

**Public Health Implications—What are the key implications or messages for practitioners, policy makers and/or researchers in public health?**

- As more Two-Spirit and Indigenous LGBTQIA+ communities age, it is important to create and foster research that is inclusive, equitable, and responsive to their needs, recognizing the unique impacts of colonialism on healthy aging.
- Two-Spirit and Indigenous LGBTQIA+ communities provide unique opportunities to learn about inclusive, equitable, and expansive healthcare for everyone.

**Abstract:**

Research on Two-Spirit (2S) and Lesbian, Gay, Bisexual, Trans, Queer, Intersex, Asexual and other identities (LGBTQIA+) Indigenous communities and age-related cognitive decline (ARCD) is still an emerging field of study. Historically, Indigenous and 2SLGBTQIA+ individuals are underrepresented in healthcare research and practices. Our research question was as follows: what is the scope, breadth, and depth of published and gray literature about First Nations, Métis, and Inuit 2SLGBTQIA+ people’s experiences of aging and dementia? This scoping review used an Indigenous-informed methodology, grounding our research in a guidance committee comprising all Two-Spirit knowledge-keepers, community advocates, and scholars. This method adapts a five-step scoping review approach, including Indigenous knowledge through consultation with Indigenous community members. The committee informed all five steps of the scoping review methodology. Our initial search identified 1320 articles; after screening, seven articles remained, comprising six journal articles and one book chapter. Manuscripts were published in Canada, the USA, and Australasia. There were five qualitative studies, one scoping review, and a book chapter. The aims, results and recommendations from the included studies are presented. We found minimal published literature on the intersecting identities of 2SLGBTQIA+ Indigenous Peoples and ARCD. Gaps included epidemiological research, assessment and interventions, and qualitative experiences in this population. Further investment in research is needed to expand what is known to understand the needs of Indigenous 2SLGBTQIA+ people with dementia.

## 1. Introduction

Age-related cognitive decline (ARCD) continues to be a growing concern among all populations, but research on ARCD and Two-Spirit people is largely absent. The existing research on Two-Spirit people and ARCD is limited and does not adequately differentiate the experiences of Two-Spirit (2S) people from non-Indigenous people who identify as lesbian, gay, bisexual, transgender, queer, intersex, asexual, or other identities (LGBTQIA+). Two-Spirit is not a universal definition but rather an umbrella term for Indigenous people who have been described as possessing both masculine and feminine spirits [1].

Two-Spirit people can be conceptualized as the merging of gender, sexuality, and spirituality that hold cultural significance as activists, knowledge-keepers, and Elders [1,2]. Two-Spirit people are of diverse gender identities (including trans, non-binary, and other genders), community roles and sexual orientations (including various same-sex- or multiple-gender-attracted, gay, bisexual, pansexual, lesbian, and other sexualities) that exist outside of the cis-heteronormative gender binary. Not all Indigenous people who identify as queer also identify as Two-Spirit [1]. Deeper insights into the definitions and roles of Two-Spirit people vary between Nations and communities; often, Two-Spirit people assume roles as healers or spiritual leaders [1].

The term “Two-Spirit”, derived from the Anishinaabe word “*niizh manidoowag*”, was introduced in a ceremony in 1990 by Elder Myra Laramee; however, the concept has existed since time immemorial [3]. One of the devastating impacts of colonization has been the imposition of the colonial gender binary on Indigenous communities, as it attempted to erase the contributions and place that Two-Spirit people hold in communities. Restrictive understandings of gender and sexuality have also led to queerphobia that Two-Spirit people may experience throughout their lives and notably in the healthcare system. Two-Spirit people have had to navigate queerphobia as well as anti-Indigenous racism in the healthcare system; this has affected the quality and accessibility of the health services that Two-Spirit people receive [1].

Colonization has had a significant effect on the health and well-being of Two-Spirit people, and its cumulative effects are apparent in the rates of ARCD in Indigenous and queer communities. Age-related cognitive decline (ARCD) refers to the gradual, non-pathological reduction in cognitive functioning associated with normal aging, particularly in domains such as memory, processing speed, and executive functioning, while other abilities such as semantic knowledge may remain relatively stable [4,5]. For the purposes of our review, we also included dementia, Alzheimer’s disease, and other cognitive problems associated with aging [6]. The prevalence of dementia is expected to increase by 187% by 2050 for the general population in Canada, but 230% for Indigenous men, and 300% for Indigenous women [6]. Colonization is at the root of the anti-Indigenous racism, cumulative stress and intergenerational trauma that not only causes poor health but prevents adequate care from being received. Indigenous people in both rural and urban areas experience the contemporary and ongoing effects of colonialism. Indigenous communities accessing rural health services often experience difficulty receiving timely specialized care services. There are also challenges in urban areas when it comes to receiving proper healthcare, as urban health services are less likely to provide culturally safe care. The lack of accessible and culturally safe health services affects the rates of dementia and age-related cognitive decline detection and intervention in Indigenous communities, as it impacts people’s ability to receive preventative care, diagnosis and treatment [7].

To address the rising rates of ARCD in Indigenous communities, an increasing amount of Indigenous-led research has emerged with culturally responsive tools. The Canadian Indigenous Cognitive Assessment (CICA) is an example of a culturally safe and accurate way of detecting cognitive impairment in Indigenous older adults [8]. ARCD places Indigenous older adults in a vulnerable position, but centering holistic Indigenous worldviews and cultural safety can help to mitigate the power imbalance in healthcare [8]. Despite the challenges, Two-Spirit people continue to be resilient and honor their roles in community [1]. This scoping review sought to answer the following research question: what is the scope, breadth, and depth of the published and gray literature about First Nations, Métis, and Inuit 2SLGBTQIA+ people’s experience of aging and dementia?

### Research Team Positionality

The team of researchers participating in this scoping review comprises individuals with diverse identities and social positions.

KD King is a citizen of the Otipemisiwak Métis Government in Alberta and a proud *ayahkwew* (2-spirit) registered nurse and researcher from northern Alberta, Canada. Their experiences as a queer and gender-diverse nurse and researcher, as well as a community member, pipe carrier, and *oskapiew* (helper) to *Nehiyaw* (Cree) and Métis Elders, inform the relational ways in which research is conducted with our research team and the other-than-human kin who support this work.

Skye Wilson is Two-Spirit from the Gitxsan Nation, studying Indigenous literature and working on a Master of Arts in English at the University of Northern British Columbia. Skye has held various roles supporting 2S/LGBTQIA+ communities, but now focuses on the health and wellness of Two-Spirit people. Skye understands the importance of community-led initiatives that expand networks of care and support, strengthening and affirming all LGBTQIA+, Indigiqueer, and Two-Spirit individuals. With thoughts of relatives, Elders, and ancestors, Skye is committed to closing the cyclical gaps in care related to brain health and Elder care and to affirming all identities we may carry in this lifetime.

Letebrhan Ferrow is a Black woman of Eritrean descent. She is an MPH graduate and is guided by a commitment to advancing health equity. Letebrhan lives and works on One Dish Wampum Territory in what is now Ontario, Canada.

Lane Bonertz is a Two-Spirit member of the Piikani Nation, using his lived experience to contribute to health research and resource development that centers Indigenous identities, cultures, and experiences. Currently, Lane is the Associate Director of Two-Spirit Health at the Community-Based Research Centre (CBRC). He supports several initiatives focused on cultural revitalization and storytelling, ensuring that the stories of our ancestors are preserved for future generations. Lane has worked and volunteered in various roles in 2SLGBTQ+ advocacy, as well as HIV and sexually transmitted blood-borne infection (STBBI) treatment and prevention. Growing up in rural Alberta, Lane’s advocacy approach is shaped by his background in ranching, agriculture, and small-town life. His contribution to this project is motivated by the love he has for his grandmother, who is in the early stages of dementia. Lane is currently based in Montréal/Tiohtià:ke, Québec, Canada.

Jennifer Walker (she/her) is a member of the Six Nations of the Grand River and lives in Hamilton, Ontario. She is a cisgender woman with Haudenosaunee and mixed European ancestry. She is an epidemiologist and aging researcher.

Megan Kennedy (she/her) is a cisgender, white settler woman of Ukrainian and Irish descent. Her perspective is shaped by these identities, which situate her outside of both Indigenous and LGBTQIA+ communities and limit her lived experience and understanding of the health needs of Indigenous LGBTQIA+ populations. In supporting this review as a librarian and expert searcher, she strove to remain reflexive about these dynamics, to acknowledge the potential blind spots they create, and to center the voices, experiences, and scholarship of Indigenous and LGBTQIA+ communities.

## 2. Materials and Methods

As a group of researchers with diverse genders and sexualities, we drew on Indigenous kinship relationality [9] in conceptualizing the review process and methods. In addition, Indigenous community engagement, as is proposed in Phillips-Beck et al. [10], occurred through visiting with our relations within the Two-Spirit dementia guidance committee and the Two-Spirit Program at the CBRC and with our colleagues at the universities. Through this collaborative process, we were able to choose the scoping review method, map out our search terms, and agree on a consensus-based approach to managing conflicts through consensus meetings to decolonize the process outlined below.

In this scoping review, we employed the adaptation of Arksey and O’Malley’s [11] five-step approach by Phillips-Beck et al. [10]. This methodology enabled us to map the extent, range, and nature of literature in the topic area while identifying gaps and opportunities for further research [12]. The five stages of this review included: identifying the research question; identifying relevant studies; selecting studies; extracting and charting data; and collating, summarizing, and reporting the results [11,12]. Each stage of the review process included consultation with the 2Spirit Advisory Committee and research team. A health sciences librarian (MK) conducted a comprehensive systematic search in November 2024. The following bibliographic databases were searched from inception to 13 November 2024: Medline (1946–present), EMBASE (1974–present), and PsycINFO (1806–present) via OVID; Cumulative Index to Nursing and Allied Health Literature (CINAHL; 1936–present), HealthSource: Nursing/Academic Edition (inception–present), and LGBTQ+ Source (inception–present) via EBSCOhost; and Scopus (1976–present). To enhance search sensitivity, databases were searched using a combination of subject headings (controlled vocabularies), such as Medical Subject Headings (MeSH), and natural language keywords. The search strategies used were structured using three main concepts: (1) 2SLGBTQIA+ community; (2) Indigenous populations worldwide; (3) aging, dementia, or elderly population. For the full search strategy for each database, please refer to Supplementary Material S3. No study type, publication date, or language limits were applied to the search results.

Results were exported from the databases in complete batches and imported into the synthesis review software, Covidence^TM^ (https://support.covidence.org/help/how-can-i-cite-covidence, accessed on 12 January 2026) [13]. This software removed duplicate records, managed title/abstract and full-text screening, and facilitated data extraction. This study was conducted and reported according to the Preferred Reporting Items for Systematic reviews and Meta-Analyses extension for Scoping Reviews (PRISMA-ScR) Checklist [14] (see Supplementary Material S2).

### 2.1. Inclusion Criteria

Inclusion criteria were determined using the Population, Concept, and Context (PCC) framework [15] (full inclusion and exclusion criteria are in Table 1). Our focus population was “Indigenous Two-Spirit, Lesbian, Gay, Bisexual, Trans, Queer, Intersex, Asexual and additional identities (2SLGBTQIA+)”; the concept was “dementia” or “age-related cognitive decline”; the context was “former British Settler-states”, including Canada, the United States of America, Australia, New Zealand, or any other settler–colonial state. We chose to use this context as these settings reflect the unique colonial experience imposed by British Imperial Settler Colonization, whereby the colonial project of Indigenous displacement, replacement, and genocide was enacted, rather than the other forms of colonization that exist in many parts of the world [16]. It was most relevant to the exploration of gender and sexually diverse Indigenous Peoples in Canada, where the researchers are located and hoping to apply the learnings gleaned from the review. We sought peer-reviewed journal articles published in English or French, the two official languages of Canada. There were no time limits on when articles were published. We also searched the gray literature, including theses, dissertations, books, book chapters, and unpublished reports, on the websites of federal and provincial/territorial health departments. We excluded theoretical discussions, editorials, opinion pieces, and conference abstracts. We also excluded literature focused solely on either Indigenous people or LGBTQIA+ people, because these do not directly reflect the intersectional identities relevant to our research questions.

### 2.2. Study Selection

Two reviewers, with at least one Two-Spirit reviewer in each case, independently screened titles and abstracts for inclusion, followed by full-text screening using the inclusion and exclusion criteria described in Table 1. Any discrepancies were resolved through discussion between the team of reviewers. Quality appraisal was not conducted, as this review focused on mapping the breadth and depth of the existing literature rather than synthesizing the studies’ findings [11,12]. Once full-text screening was completed, the included studies were exported into an MS Excel spreadsheet for data extraction and analysis.

All studies were extracted by a primary and a secondary reviewer, at least one of whom was Two-Spirit in each case, who verified the extracted data. The reference lists of all studies were hand-searched for additional research to include. Data extracted included study details (title, authors, publication year, journal, DOI), country of study, discipline of journal published in, research aim/purpose/question, study design (qualitative/quantitative/mixed methods), specific methods, data sources, results/outcomes, and recommendations for further research. Reviewer comments were also collected. Data was charted in additional MS Excel spreadsheets, and pivot tables were used to analyze numerical data. Narrative data were analyzed by two reviewers and are described below.

## 3. Results

**Figure 1:**
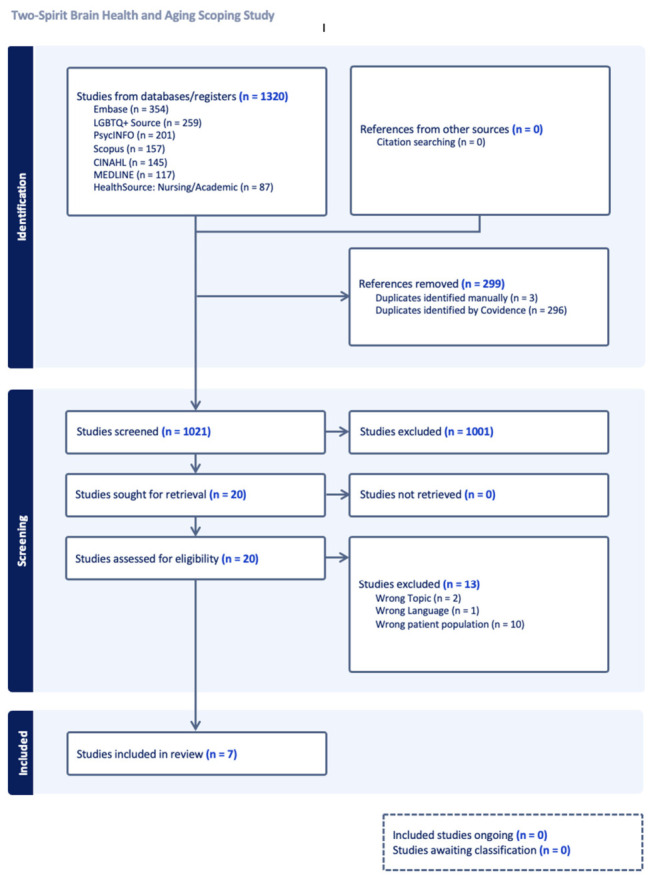
Prisma Scoping Review Diagram.

The search strategy yielded 1320 articles that included our search terms. We identified and removed 299 duplicates (three manually and 296 by Covidence). Title and abstract screening were completed for 1021 articles, and 1001 studies were removed for not meeting the inclusion/exclusion criteria. The remaining 20 studies were retrieved, and full-text screening was completed, leaving seven studies for data extraction and analysis (for the full Data Extraction Table, see Supplementary Material Data S1). In both title and abstract screening and full-text screening, the majority of articles were excluded because they did not include all of the criteria of interest (e.g., Indigenous Peoples + ARCD, without 2SLGBTQIA+, or Indigenous Peoples and 2SLGBTQIA+, without ARCD). See Figure 1 for the Prisma Scoping Review diagram.

The characteristics of the seven studies are described here. The included studies were published between 2010 and 2024, with four studies appearing in journals related to aging, one in a social sciences and health journal, one in an LGBT aging book, and one in *Alzheimer’s & Dementia* journal. There were three studies from Canada, three from the United States of America (USA) and one from Australasia. Of these studies, five were qualitative, one was a book chapter, and one was a review article. Specific methods of enquiry included narrative (n = 3), digital storytelling (n = 1), scoping review (n = 1), case study (n = 1), and policy analysis (n = 1). Various data sources were used to inform the research studies included; see Table 2 for complete details.

From the included studies, the researchers’ aims varied widely. Research suggests that emphasizing individual experiences with dementia can enhance our understanding of how various intersecting identities are affected by dementia [17]. Tovey [18] reinforces this concept, especially at the crossroads of race and sexuality, by illustrating the systemic damage of colonialism through the experiences of Black LGBTI individuals in Australia. Addressing the historical oppression, healthcare barriers, and policy challenges confronting marginalized communities can be achieved by incorporating Two-Spirit values, knowledge, Elders, roles, and traditions [19]. Furthermore, Chazan [20] highlights the practices of Indigenous women as a concept of moving beyond the normative practices of care. Understanding cultural safety as a vital strategy for enhancing services and dementia care for Indigenous Peoples was the aim of Chakanyuka et al.’s [21] review. Trinh [22] enhances these discussions by integrating ageism and ableism with race, sexual orientation, and gender identity. Maestre [23] highlights health disparities that primarily affect Two-Spirit and LGBTQIA+ Indigenous groups.

The research findings of the included studies were as diverse as their research aims. O’Connor [17] described three dominant themes in an Indigenous lesbian woman’s experience of being diagnosed with dementia. The first theme explored a desire to connect with culture, as this helped her reinterpret the effects of cognitive changes to get closer to her ancestors. The second theme explored the ways her interpretation of dementia, her age, and atypical presentation resulted in a discounting of the impact of dementia on her life. The last theme explored the lack of recognition (by healthcare systems) of her same-sex partner, which resulted in isolation and relationship challenges. Tovey [18] highlights institutional barriers that gay men experience and provides context around the racial and homophobic discrimination embedded in institutions, as well as the importance of advocacy around aging with dignity, holistic care that acknowledges and improves safety for LGBTQI people. Harley [19] provides a chapter that discusses the history of Two-Spirit people across various Indigenous communities in North America, highlighting the diverse health outcomes for Two-Spirit individuals as a minority within a minority. Chazan [20] critiques the heteronormative and ableist conceptions of aging, with the emphasis placed on reproduction and self-sufficiency. This Indigenous perspective challenges colonial perspectives on heteronormative family structures and the value of older people, as a form of colonial violence against Indigenous kinship networks, highlighting intergenerational relationships as central to healthy aging.

In Chakanyuka’s [21] scoping review, the authors identified 17 articles on cultural safety in dementia care for Indigenous people from Canada, Australia, and New Zealand. Indigenous cultural safety was discussed on three levels: individual, healthcare provider, and organizational, with only one study identifying sex, gender, sexual orientation or gender expression or identity as important to cultural safety. Trinh [22] broadly discusses the intersectionality that older adults embody, including sexuality, gender, indigeneity, and others; however, they do not specifically explore the experiences of Two-Spirit Indigenous people. Maestre [23] highlights the need for greater inclusivity and diverse representation in dementia research. This would assist researchers in understanding the risks, resilience, and protective factors operating in diverse communities, thereby contributing to the safety and efficacy of trials. The study suggests a better application of a specific health disparity research framework to better understand disparities related to aging.

**Table 2:** Characteristics of Included Studies.

First Author	Year	Title	Journal/Book	Discipline of Journal	Country	Study Design	Specific Methods	Data Sources
Maestre [23]	2024	Promoting diverse perspectives: Addressing health disparities related to Alzheimer’s and all dementias	*Alzheimer’s & Dementia: The Journal of the Alzheimer’s Association*	Alzheimer’s and Dementia	USA	Qualitative	Narrative Summary	Meeting summaries
Trinh [22]	2023	Reversing the Systemic Tide to Truly Lift All Boats	Generations	Aging	USA	Qualitative	Policy Analysis	Government agencies
Chakanyuka [21]	2022	Indigenous-specific Cultural Safety within Health and Dementia Care: A scoping review of reviews	*Social Science & Medicine*	Social Sciences and Health	Canada	Review	Scoping Review	Peer-reviewed review articles
Chazan [20]	2020	Unsettling Aging Futures: Challenging Colonial-Normativity in Social Gerontology	*International Journal of Ageing and Later Life*	Aging	Canada	Qualitative	Digital Storytelling	Interviews, oral history, storytelling
Harley [19]	2016	American Indian, Alaska Native, and Canadian Aboriginal Two-Spirit/LGBT Elderly	*Handbook of LGBT elders: An Interdisciplinary Approach to Principles, Practices, and Policies*	LGBT Aging	USA	Book Chapter	Narrative	Multiple sources, book chapter
Tovey [18]	2015	Little Black Bastard	*Australasian Journal on Ageing*	Aging	Australia	Qualitative	Narrative	Personal story
O’Connor [17]	2010	Dementia at the intersections: A unique case study exploring social location	*Journal of Aging Studies*	Aging	Canada	Qualitative	Case study	Interviews, video-taped participant observation

Table 3 highlights the main recommendations from the included studies. These recommendations from the studies offer valuable insights, helping us better understand the gaps in dementia healthcare and the resources available to address them. First, research focused on the lived experiences of individuals with dementia offers valuable insight into the diversity and complexity of these experiences, allowing us to genuinely hear their voices [17]. The O’Connor case study [17] examines different dimensions of social isolation related to dementia, considering all elements of identity as influencing factors, thereby highlighting the socio-cultural context. Tovey [18] argues that healthcare practices can be enhanced through social investments, emphasizing the cultural differences between medical institutions and racialized or LGBTI communities. Advocacy for community-based discussion to identify culturally relevant and safer care for Indigenous gender-diverse individuals for meaningful conversations about sex, sexuality, gender identity, and LGBT issues that are contextualized in colonialism [19]. Ongoing colonization, such as heteronormativity and gender roles, cumulatively impact ongoing trauma, which supports further programming for health and mental health practices that are safer for Two-Spirit and LGBTQ Indigenous people [19]. Community is an important support system for Two-Spirit and Indigenous communities [19]. Additionally, it is essential to recognize that aging is not a linear process, particularly in the context of evolving identities, as all stages of life hold significance in Indigenous communities [20]. Studies of Indigenous and 2SLGBTQIA+ communities should identify measures to evaluate and assess cultural safety in healthcare delivery. This can help improve the quality of evidence and inform future health policy, planning, and practices. These improvements redress the legacy of colonialism and racism in healthcare [21]. Practices of diversity, equity, and inclusion are crucial across all social institutions, especially in healthcare for older adults with intersecting identities [22]. Researchers, sponsors, and regulatory bodies are increasingly involving more diverse populations in dementia research. However, this necessitates innovative approaches, creative recruitment strategies, distinct methodologies, varied research teams, and accountability in all processes [23].

## 4. Discussion

Our scoping review identified only seven studies that met the inclusion criteria, highlighting a distinct lack of research on the intersections of Indigeneity and 2SLGBTQIA+ with age-related cognitive decline. In addition to this, all the included studies were from settler-states from high-resource countries, including Canada, the USA, and Australia, which suggests an even further lack of acknowledgement of Indigenous and queer realities in research from lower-resource settings. There is a clear opportunity to expand research and further explore this unique sub-population of adults who experience ARCD in diverse social and cultural environments.

Five of the seven studies included in our review were qualitative, comprising one review and a book chapter, reflecting the limited availability of quantitative research in our Indigenous and 2SLGBTQIA+ communities. Diverse methods were used in the included studies, and the data sources were similarly diverse; however, testimony from 2SLGBTQIA+ Indigenous people was the most common. Across the studies in our scoping review, themes of displacement, lack of access, and poorer health outcomes were prevalent. Additionally, connection to Indigenous culture, intergenerational learning, and representation of Indigenous 2SLGBTQIA+ people were highlighted as helpful in addressing exclusion. There were distinct gaps in the literature regarding epidemiological data for Indigenous 2SLGBTQIA+ people experiencing cognitive changes related to aging, as well as gaps in Nation-specific or distinction-based qualitative research, which is necessary to account for the vast diversity of the Indigenous experience and life. In the discussion below, we will relate our findings to other research on cognitive decline, dementia, and aging.

Epidemiological data on dementia are often aggregated by demographic groups. For example, the Alzheimer’s Society of Canada’s Landmark Study presents statistics that are limited to Indigeneity, ethnicity, sex, and age [6]. Dementia rates that incorporate Two-Spirit identity, sexual orientation and diverse gender identity are not often included due to the limitations of population-level datasets available [6]. This research gap limits our understanding of the scope and complexity of aging among Two-Spirit older adults. Recent progress has been made with the Canadian Longitudinal Study on Aging (CLSA), a national cohort study of over 50,000 participants. The CLSA began recruiting participants in 2010 and plans to follow participants until 2033, collecting data through questionnaires and physical assessments every few years [23]. A question on Two-Spirit identity has been added to the CLSA questionnaire for the Follow-up 4 data collection [24,25]. Including this question can provide empirical evidence to support Two-Spirit researchers and advocates in their efforts to support 2SLGBTQIA+ Indigenous older adults as they age.

The available literature suggests, as expected, that 2SLGBTQIA+ and Indigenous populations face unique challenges in healthcare, which are further complicated by aging and the associated cognitive decline or dementia experienced by these communities. Historically, these communities have faced discrimination and mistrust in healthcare systems [17]. As a result, marginalized communities rely entirely on their networks to provide care [18]. While existing research focuses on individual experiences and their causes, studies on Indigenous and 2SLGBTQIA+ groups suggest that specific social programs require further research and development [19]. These studies indicate that an increased implementation of inclusive and trauma-informed care is needed to support Two-Spirit, Indigenous and LGBTQIA+ people experiencing cognitive decline due to aging [19].

Furthermore, housing and care facilities for these specific populations are identified as a need [19]. Still, it is essential to recognize that every Indigenous community and gender/sexual orientation has unique needs and experiences. Another challenge to consider is the geographical location of care facilities. For individuals living in rural areas, relocating to urban centers can be difficult, as it removes them from their respective lands and communities [19]. It is recommended that more community-led 2SLGBTQIA+ programming and education be made available for Indigenous communities, ensuring that Two-Spirit individuals do not have to leave their communities if they do not wish to [20]. This approach fosters community interconnectedness, enhances cultural safety and provides opportunities for communities to define and frame their work in ways that reflect their unique strengths, needs and opportunities. Community-led education on gender and sexuality is proposed as an effective means to contextualize sex, sexuality, and gender diversity through a decolonial lens, which subsequently strengthens communal relationships in rural areas. As recommended in the studies above, there is a need for inclusive, culturally relevant, and safer spaces for Two-Spirit and LGBTQIA+ folks living in rural areas. This aligns with investments in community development, including support for the elderly, which emphasizes community-led initiatives. The studies above affirm community-based care by conceptualizing aging as a nonlinear, cyclical process; this conception of the lifecycle values the lives of our elderly rather than devaluing aging. 2SLGBTQIA+ and Indigenous communities can benefit from education on aging, sex, gender, and sexual orientation, with a focus on community [20].

We were unable to find any assessment for cognitive decline that acknowledges the unique lived experiences of 2SLGBTQIA+ Indigenous people. While the CICA is an example of a culturally safe and accurate way of detecting cognitive impairment in Indigenous older adults [8], it does not go far enough to explore the unique needs of 2SLGBTQIA+ Indigenous adults, whose life experiences may differ from those of their cis-heterosexual counterparts. Further research is warranted to understand the complexity of gender-affirming healthcare on ARCD [26], as well as other health-related factors (i.e., HIV status, exposure to highly active antiretroviral therapy, minority stress) [27], and other intersectional and socially determined influences on health.

Research on dementia and cognitive decline should begin with conversations involving the Two-Spirit, LGBTQIA+, and Indigenous communities, as these groups face distinct needs and access points. Engaging with these populations can help contextualize cultural relevance, enhance safety, and inform policy, research, and care practices [21]. This scoping review highlights the lack of attention given to gender and sexually diverse Indigenous populations as they age. Preparations and planning for these communities as they age can only improve the existing healthcare practices to be more inclusive and holistic. As mentioned above, aging should be understood as cyclical to better support elderly Indigenous people. The studies above suggest that our research, policies, and funding should prioritize promoting equity, diversity, and inclusion, particularly for diverse older adults, including 2SLGBTQIA+ Indigenous adults [20,27].

### Strengths and Limitations

The strengths of our scoping review include the support of our Two-Spirit Advisory Committee, who were essential to all aspects of the project. In addition, the contributions of a research librarian knowledgeable in Indigenous research and cognitive research were foundational in developing our search strategy and terms, as well as our inclusion and exclusion criteria. The research team is composed mainly of Two-Spirit researchers from various disciplines and Indigenous communities, which provides additional rigor and credibility to our process. All articles were reviewed by at least one Two-Spirit researcher, and the manuscript was prepared in accordance with the Preferred Reporting Items for Systematic reviews and Meta-Analyses extension for Scoping Reviews (PRISMA-ScR) Checklist.

The limitations of our scoping review were minimal. We attempted to include a broad range of studies across languages, but ultimately excluded papers not in English or French (both official languages in Canada). However, it is possible that papers published in other languages were not captured in our search, as we primarily searched English-language databases using English-language search terms. Unfortunately, these are the most common scientific databases for our academic institutions. Our search strategy was as comprehensive as possible; however, there may be words in Indigenous languages or terms that are used in other contexts to describe queer or Indigenous or 2SLGBTQIA+ Indigenous people. Additionally, it is possible that these limitations in search terms, as well as potential structural under-indexing (for example, Two-Spirit being subsumed into LGBT or other variants of the acronym in databases) led to under-representation of the topics in the search. We attempted to be as inclusive as possible in our inclusion and exclusion criteria and used dual review for data extraction and validation to minimize errors and omissions. One limitation may be that we did not expand the Arksey and O’Malley [11] method to include expert review, which may have led to the identification of additional studies or the derivation of further insights from the findings. However, by integrating input from the Two-Spirit Advisory Committee, the project included community input.

## 5. Conclusions

Our scoping review outlines the scope, depth, and breadth of the published literature in English and French related to experiences of 2SLGBTQIA+ Indigenous Peoples and ARCD or dementia. We have found very limited research that addresses the intersectional nature of 2SLGBTQIA+ Indigenous people’s identities of older adults, as well as limited research outside of high-resource settler–colonial states. Several dementia studies explore either Indigenous or 2SLGBTQIA+ identities, but very few address the experience of both. We found no quantitative data on the incidence or prevalence of cognitive changes for aging 2SLGBTQIA+ Indigenous people. This lack of epidemiological data is a significant gap that has the potential to undermine the quality of life and provision of services for those who experience cognitive decline. The qualitative literature broadly described the experiences of cognitive decline and challenges faced by some 2SLGBTQIA+ Indigenous people; however, there was a lack of representation from the diverse nations that exist around the world. There was a lack of strengths-based and community-driven research to inform assessment, treatment, and service planning and delivery for this unique population.

Based on the findings of our scoping review, additional research is needed to further explicate the quantitative and qualitative aspects of 2SLGBTQIA+ Indigenous Peoples’ experiences with ARCD and the associated care needs. There is a clear need for epidemiological research to identify the incidence and prevalence of ARCD in Two-Spirit communities. Additional cross-sectional or longitudinal intersectional research is needed to further understand the life course of cognitive decline and diverse care needs for 2SLGBTQIA+ Indigenous Peoples as they age. Qualitative research is also necessary to gain a deeper understanding of the diverse experiences and interactions with the health system that 2SLGBTQIA+ Indigenous people encounter. This research should be community-led and center Indigenous epistemologies, ontologies, cosmologies, knowledge systems, and research methodologies and methods. Adequate funding and support are required to build community capacity, supporting nation-specific, self-determined research priorities and processes.

## Figures and Tables

**Table 1:** Study Inclusion and Exclusion Criteria.

Criterion	Inclusion	Exclusion
Population	Indigenous Peoples Indigenous, First Nations, Métis, Inuit, American Indian, Alaskan Native, Native Hawaiian Topic of paper related to Two-Spirit (or Indigenous LGBTQIA+) Two-Spirit, Indigiqueer, lesbian, gay, bisexual, trans, queer, intersex, asexual Any study that includes adults (aged 18 years or older) Human studies	Non-Indigenous Cisgender or straight Studies whose primary focus is children (under 18 years) Studies on animals or non-human relations Studies in languages other than English and French (without a translation available)
Exposure	Dementia or aging Brain health Cognition Dementia Aging Alzheimer’s	Studies unrelated to aging or dementia
Context	Anywhere in the world Any time published	N/A

**Table 3:** Summary of Study Recommendations.

Author (Year)	Recommendations:
O’Connor [17], 2010	Qualitative studies are preferred to actually ‘hear’ the experiences of those living with dementia. Social location heavily influences 2SLGBTQIA+ and Indigenous experiences [considering identity] and shapes their access to healthcare significantly.
Tovey [18], 2015	A recommendation is to learn about the additional community care practices unique to LGBTI and racialized groups. Social isolation is a concern with LGBTI groups, reallocating time and attention to intentional social spaces.
Harley [19], 2016	General suggestions for Indigenous seniors: Culturally responsive programming and employment in healthcare systems; Coordinated an elderly care funding initiative for Aboriginal caregivers; Aboriginal long-term care facilities in the major prairie cities; An integrated, coordinated, and holistic healthcare system; First Nations long-term care facilities on reservations; Palliative, respite and after-hours care services; Access to all health benefits. Specific recommendations for Two-Spirit and LGBTQIA+ seniors: Specific actions, such as an increase in community support and integration of traditional culture and healing practices, are needed to address the specific needs of Aboriginal gender-diverse people. Community consultations to identify culturally relevant and meaningful ways to discuss sex, sexuality, gender identity and LGBT issues. Contextualizing homophobia within the context of colonization, the historical trauma, and the cumulative violence Two-Spirit identities experience. Developing specific mental health and safer spaces for Two-Spirit people, especially Youth. Public policies should encourage the government to take a greater responsibility for long-term care, with increased support for families. Wholistic Aboriginal Elder care: includes culture, community, and mixed living systems—promoting community as integral to their healthcare.
Chazan [20], 2020	Aging is a nonlinear process, especially in terms of a person’s evolving identities. Understanding lifecycles as nonlinear helps challenge notions of older Indigenous adults having ‘less value,’ and instead suggest they having thriving futures.
Chakanyuka [21], 2022	Research should identify specific measures to evaluate and assess cultural safety, which could help improve the quality of evidence, policy, planning, and practice.
Trinh [22], 2023	Future policies should promote equity, diversity, and inclusion, especially for older adults with intersecting identities.
Maestre [23], 2024	Research, study sponsors and regulatory agencies should seek better engagement with these populations. New approaches to funding, innovative recruitment strategies, diverse research teams and accountability.

## Data Availability

No new data were created or analyzed in this study.

## Supplementary Materials

The following supporting information can be downloaded at: https://www.mdpi.com/article/10.3390/ijerph23060735/s1, Supplementary Material S1: Data Extraction Table; Supplementary Material S2: PRISMA-ScR Checklist; Supplementary Material S3: Search Strategy: Indigenous 2SLGBTQIA+ Dementia and Aging.

## Abbreviations

The following abbreviations are used in this manuscript:

2SLGBTQIA+	Two-Spirit Lesbian Gay Bisexual Transgender Queer/Questioning Intersex Asexual/Ally and any other identities not described.
ARCD	Age-Related Cognitive Decline
PRISMA-ScR	Preferred Reporting Items for Systematic Reviews and Meta-Analyses extension for Scoping Reviews
CLSA	Canadian Longitudinal Study on Aging
PCC	Population, Concept, and Context
